# A Polymorphic (CT)*n*-SSR Influences the Activity of the *Litopenaeus vannamei IRF* Gene Implicated in Viral Resistance

**DOI:** 10.3389/fgene.2019.01257

**Published:** 2019-12-06

**Authors:** Bin Yin, Haiyang Wang, Peng Zhu, Shaoping Weng, Jianguo He, Chaozheng Li

**Affiliations:** ^1^Southern Marine Science and Engineering Guangdong Laboratory (Zhuhai)/School of Marine Sciences, Sun Yat-sen University, Guangzhou, China; ^2^State Key Laboratory of Biocontrol/School of Life Sciences, Sun Yat-sen University, Guangzhou, China; ^3^Guangxi Key Laboratory of Beibu Gulf Marine Biodiversity Conservation, Beibu Gluf University, Qinzhou, China

**Keywords:** computed tomography (CT) microsatellite, *Litopenaeus vannamei IRF*, white spot syndrome virus (WSSV) resistance, molecular marker, *Litopenaeus vannamei*

## Abstract

Simple sequence repeats (SSRs) of short nucleotide motifs occur very frequently in the 5′ untranslated coding region (5′-UTR) of genes and have been implicated in the regulation of gene expression. In this study, we identified an SSR with a variable number of CT repeats in the 5′-UTR of the *Litopenaeus vannamei IRF* (*LvIRF*) gene that has been shown to mediate antiviral responses by inducing the expression of Vago, a functional homolog of mammalian IFN. We then explored the effects of varying the number of (CT)*n* repeats on the expression of *LvIRF* using both dual-luciferase reporter assays and Western blots. Our results demonstrate that the length of the (CT)*n*-SSR in this gene can influence the expressional level of *LvIRF*, in that a shorter (CT)*n* repeat had a stronger ability to induce the expression of *LvIRF*. Moreover, we found that the (CT)*n* repeat in *LvIRF* was associated with viral resistance in shrimp. Individual shrimps with shorter (CT)*n* repeats in the 5′-UTR of *LvIRF* exhibited high tolerance to white spot syndrome virus (WSSV), and this trait was inherited in offspring. Taken together, these results indicated that this (CT)*n*-SSR could be used as a molecular marker for shrimp breeding for WSSV resistance.

## Introduction

Simple sequence repeats (SSRs), also known as microsatellites or short tandem repeats (STRs), are tandem repeats of short sequence motifs occurring ubiquitously in eukaryotic genomes. They exhibit extensive polymorphism due to variations in the copy number of each specific repeat motif, and are considered useful as genetic markers for genetic diversity analysis, DNA fingerprinting, and linkage mapping. Several studies have suggested that SSRs are non-randomly distributed in the genome, as untranslated coding regions (UTRs), and have more SSRs than coding regions, as well as exhibit a strong bias towards di- and tri-nucleotides repeats (Metzgar et al., 2000; Wren et al., 2000; Li et al., 2002; Morgante et al., 2002; Fujimori et al., 2003). SSRs in UTRs have been found to be associated with gene expression by affecting transcription factor binding, methylation of CpG and/or DNA structure modification (Li et al., 2004).

Some of the best-known examples of SSRs affecting gene expression come from human genetic disorders. For example, fragile X syndrome (FXS) has been attributed to the absence of fragile X mental retardation 1 (*FMR1*) gene expression due to a long CGG tri-nucleotide repeat in the *FMR1* gene 5′-UTR adjacent to the promoter that results in the *FMR1* gene’s epigenetic silencing (Colak et al., 2014). In another study, a polymorphic (CA)*n* microsatellite identified in the 5′-UTR of the prolactin 1 (*prl 1*) gene of tilapia has been associated with differences in *prl 1* gene expression and the growth responses of salt-challenged tilapia (Streelman and Kocher, 2002). In *Drosophila*, a series of polymorphic (GA)*n* microsatellite sequences in promoter regions have been shown to bind to a protein family called GAGA factors, which are involved in gene expression (Tsukiyama et al., 1994; Berger and Dubreucq, 2012). In mammalian cells, three novel downstream elements, which show homology to GAGA factor binding sequences, can regulate promoter activity and preferentially affect transcription start site (TSS) selection at the 5′-UTR end of promoters (Lee et al., 2010).

Disease resistance is a low heritability trait that is easily influenced by external environments. Designer breeding using molecular markers is considered to be an effective method for the cultivation of disease resistance and other complex traits in crops and livestock (Xue et al., 2013). Whole genome resources and transcriptome resources provide opportunities to dissect the genes controlling complex traits and can be used to reveal the coupling mechanisms of different genes, which in turn can contribute to the use of functional genes as molecular markers for designer breeding projects (Xue et al., 2015). In order to do this, however, a thorough understanding of the functional genes related to disease resistance is needed.

The shrimp species *Litopenaeus vannamei* is a worldwide aquaculture species that was first introduced to China in the 1980s, and its production has increased rapidly in the 21^st^ century. However, various shrimp diseases are responsible for huge losses, especially the white spot syndrome virus (WSSV) (Flegel, 2012; Li et al., 2019b). To date, there is no effective method to prevent WSSV infection in shrimp, but selective breeding of WSSV-resistant species should be an effective way to solve this problem.

In shrimp, innate immunity plays a key role in the defense against a wide variety of invading microbes such as bacteria, fungi, and viruses. Several signaling pathways are essential components of innate immunity, including the Toll, IMD, and JAK/STAT pathways (Li et al., 2019a; Li et al., 2019b). In recent studies, the shrimp IRF-Vago-JAK/STAT pathway, which is similar to the IRF-IFN-JAK/STAT pathway in vertebrates, has been functionally identified to play a significant role in defense against viruses including WSSV (Li et al., 2015). The *L. vannamei* IRF (*LvIRF*) is the first interferon regulatory factor (IRF) identified in crustaceans, and has a similar protein nature as mammalian IRFs. *LvIRF* can be activated during viral infection, and then translocates to the nucleus to initiate the expression of the *L. vannamei Vago4* (*LvVago4*) gene. This is effectively an arthropod cytokine encoding a viral-activated secreted peptide through activating the JAK-STAT pathway to restrict viral infection, similar to mammalian IFNs (Chen et al., 2011; Paradkar et al., 2012; Wang and He, 2019).

The key constituents of innate immunity were regarded as molecular markers for breeding disease resistance in shrimp. In this study, we found a (CT)*n* microsatellite with a variable number of CT motifs present in the 5′-UTR of the *LvIRF* gene. We demonstrated that the number of (CT)*n* repeats modulates the promoter activity of the *LvIRF* gene in a length dependent manner, and observed that shrimp with different numbers of CT repeats showed distinct tolerances to WSSV. Furthermore, we demonstrate the use of the number of (CT)*n* repeats at this locus as a molecular marker to selectively breed a new generation of shrimp resistant to WSSV. In F2 offspring, the populations with smaller numbers of (CT)*n* repeats were more resistant to WSSV.

## Materials and Methods

### Experimental Animals and Pathogens

In order to investigate the relationship between (CT)*n* repeats and the antiviral traits of *L. vannamei*, three different populations of 100 healthy shrimp (TH01, TH03 and SP07) were collected from the Hengxing shrimp farm in Zhanjiang city, China. TH01 was collected from Vietnam, TH03 was collected from China, and SP07 was collected from Saipan. The shrimp body weight was 5.0 ± 1.0 g each, and each population of shrimp was cultured in filtered sea water with a 2.5% salinity at 26°C in a recirculating water tank and fed with fodder at rate of 5% of body weight per day. WSSV was prepared from shrimp muscle tissue previously infected with WSSV and stored at −80°C. The muscle tissue was homogenized to prepare it as a WSSV inoculum at a final concentration of 10^6^ virions/50 μl, which was injected into each shrimp (Li et al., 2014). All tanks were checked every 2 h and dead shrimp were collected and marked. On the 10^th^ day (240 h) after infection, all shrimp were collected and marked. In a study of the WSSV resistance of *L. vannamei*, the survival rate curve showed a normal distribution using this injection infection method (Huang et al., 2011). Therefore, we used mortality peaks as the cut-off point for determining resistance, and each population was divided into two groups: WSSV-susceptible and WSSV-resistant. All experimental materials were stored at −20°C with DNA holder and RNA holder (TakaRa, Dalian, China) for downstream DNA and RNA extractions.

### Analysis of Allelic Polymorphism, Promoter Sequences and Preparation of Expression Vectors

Genomic DNA was extracted from muscle tissue using the E.Z.N.A. Tissue DNA Kit (Omega Bio-tek, Doraville, GA, USA). The *LvIRF* cDNA had previously been deposited in the NCBI GenBank (KM277954), and the *L. vannamei* IRF-5′-UTR was used as a DNA template for PCR amplification (**Table 1**) (Li et al., 2015). PCR products were electrophoresed on 20% polyacrylamide gels at 80 V for 8 h in 1 × TBE running buffer (89 mM Tris-boric acid, 2 mM EDTA, pH 8.0). The polymorphic bands were purified using the E.Z.N.A. Gel Extraction Kit (Omega Bio-tek, Doraville, GA, USA), followed by cloning into the pMD-19T vector (TakaRa, Dalian, China) and sequence confirmation. The sequences were analyzed and compared using BioEdit (Hall, 1999), and those with different in length were chosen for polymorphism analysis by sequencing.

**Table 1:** The primers used in this study.

Names	Sequences (5′–3′)	Size/bp	Tm/°C
**PCR**			
LvIRF-5′UTR-F	ATCGGGATCCACTCGCAGATAC	202	56
LvIRF-5′UTR-R	GGCGACCTTAGACCGACGAGTT		
**Protein expression**			
pAc-LvIRF-5′UTR-F	GG*GGTACC*ATCGGGATCCACTCGCAGAT	202	56
pAc-LvIRF-5′UTR-R	GG*GAATTC*GGCGACCTTAGACCGACGAG		
pAc-LvIRF-F	GG*TATCCA*ATGCCGCCATCTTTCACCAATG	1086	60
pAc-LvIRF-R	GG*TCTAGA*CGGCAACGTCCTCTCGCCGGCA		
**Quantitative RT-PCR**			
LvIRF-F	ACGCTGCCCTCTTTCGCTAC	162	60
LvIRF-R	ACGCTGTGAACCTGAAGTATCG		
LvEF-1α-F	GTATTGGAACAGTGCCCGTG	143	60
LvEF-1α-R	ACCAGGGACAGCCTCAGTAAG		
**PCR for genotyping**			
LvIRF-5′UTR-F	FAM-ATCGGGATCCACTCGCAGATAC	202	56
LvIRF-5′UTR-R	GGCGACCTTAGACCGACGAGTT		

Total RNA was extracted using the RNeasy Mine Kit (Qiagen, Hilden, Germany) and reverse transcribed into cDNA using the PrimeScript RT reagent kit (TakaRa, Dalian, China). The ORF of *LvIRF* without a stop codon was used as a cDNA template for PCR amplification (**Table 1**). PCR products were then cloned into the pAc5.1-His/V5 A vector (Invitrogen, Carlsbad, CA, USA) to generate pAc-IRF-V5 and the sequence was confirmed. Different length *LvIRF*-5′-UTR sequences were then amplified from pMD-19T vectors and cloned into the pAc-IRF-V5 vector to generate pAc-IRF-(CT)*n*-V5. These were sequenced, and a pair wise alignment was then performed. The promoter regions of *LvVago4* were cloned into the PGL-3 basic vector (Promega, Madison, WI, USA) to generate a luciferase reporter gene plasmid (Chen et al., 2011).

### Dual-Luciferase Reporter Assays

To detect the effects of *LvIRF* on the promoters of *LvVago4* genes, dual-luciferase reporter assays were performed using *Drosophila Schneider* 2 (S2) cells with pAc-IRF-(CT)*n*-V5 that contained different numbers of CT repeats as IRF-expressing vectors. Cells in each well of a 96-well plate (TPP, Switzerland) were transfected with 0.05 μg PGL-*LvVago4* vector as a reporter plasmid, 0.005 μg pRL-TK renilla luciferase (Promega, Madison, WI, USA), and 0.05 μg of expression plasmid, *LvIRF* vector or empty expression vector as controls. The pRL-TK renilla luciferase plasmid was used as an internal control. 48 h after transfection the firefly and renilla luciferase activities were measured, according to the manufacturer’s instructions. Each experiment was performed in triplicate.

### Western Blot and Quantitative RT-PCR

The pAc-IRF-(CT)*n*-V5 vector was transfected into S2 cells. After 72 h, cells were harvested and lysed in NP-40 lysis buffer with protease inhibitor cocktail (Sigma, St. Louis, MO, USA). Western blot was performed with a rabbit anti-V5 antibody (Sigma, St. Louis, MO, USA) as a primary antibody, and alkaline phosphatase-conjugated goat anti-rabbit as a secondary antibody (Sigma, St. Louis, MO, USA). ImageJ was then used to measure the gray-level values and calculate the ratio of LvIRF and *β*-actin. Quantitative RT-PCR was performed for the analysis of IRF gene expression, and the EF-1α gene was detected as an internal control (**Table 1**).

### Microsatellite Genotyping

The CT microsatellite from the *LvIRF* 5′-UTR region was genotyped using FAM fluorogenic probes followed by capillary electrophoresis to discriminate allele size. Primers (**Table 1**) were designed to amplify a 202 bp fragment containing 18 CT repeats that was used as a reference, and deviation from this size allowed us to deduce the number of CT repeats for different alleles. Samples were sequenced and analyzed using an ABI Genetic Analyzer 3730 XL. We classified the alleles into two groups: short (S) alleles were ≤ 18 repeats, and long (L) alleles were > 18 repeats, based on the median (the (CT)*n* repeat numbers ranged from 13 to 24) that was adopted by referring to other reports (Gregorek et al., 2013; Dias et al., 2017).

### Establishment of HX-CTS and HX-CTL Generations

The *L. vannamei* breeding program was carried out at the Hengxing shrimp farm in Zhanjiang city, China. Choosing the HX150301 and HX1403 populations as founder stocks, two ponds were stocked with spawners, with one pond named the HX-CTS population where the parents contained short CT repeats in the *LvIRF* 5′-UTR, while the other one pond only contained long CT repeats, which we named the HX-CTL population (**Figure 5A**). When the shrimp from each pond had a body weight of 5 g, we used the artificial WSSV challenge experiment mentioned above, to draw survival rate curves.

### Statistical Analysis

Allelic frequency calculations were performed using PopGen32 software (Yeh and Boyle, 1997). We used 2 × 2 contingency tables and Fisher’s exact test to obtain *P*-values with odds ratios (ORs) and 95% confidence intervals (95% CIs) to calculate significance, as well as two-tailed unpaired *t*-tests and ANOVA. Bonferroni corrections were applied to account for the multiple testing of the CT genotype frequency [*p corr < 0.0042 (0.05/12)] and genotype groups [*p corr < 0.0167 (0.05/3)], following Fisher exact tests (Lewitter et al., 2012). ANOVA tests were followed by Tukey *post hoc* tests (*p < 0.05).

## Results

### Analysis of the (CT)*n* Variation in the 5′-UTR of the *LvIRF* Gene

**Figure 1:**
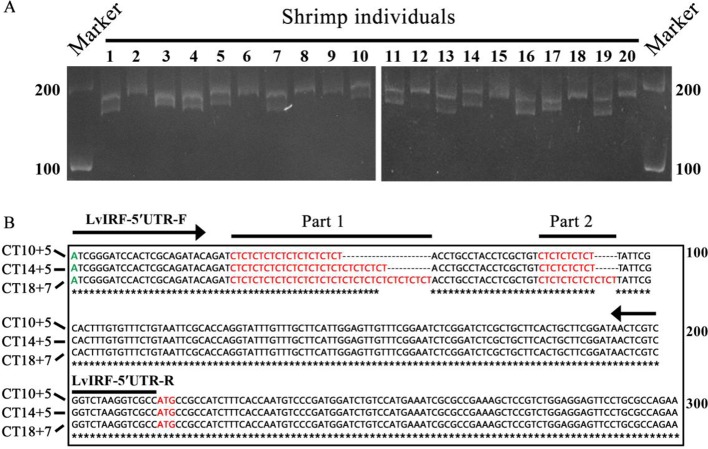
Polymorphisms in the 5′-UTR of *LvIRF*. **(A)** PCR amplified 5′-UTRs from 20 *L. vannamei* individuals were analyzed by agarose gel. Length polymorphisms between individuals was observed. The (CT)*n* repeat motifs was marked in red, transcription start site (TSS) was marked in green, and translation start site (ATG) was marked in red. The locations of primers LvIRF-5′UTR-F/R (expected size 202 bp) was shown to analysis the polymorphism of (CT)*n* repeat in shrimp. **(B)** Multiple sequence alignment of *LvIRF* 5’-UTRs. Variation in the numbers of (CT)*n* motifs, with a long (CT)*n* repeat (Part 1) and a short (CT)*n* repeat (Part 2), were distinctly observed.

LvIRF has been shown to bind the promoter of *LvVago4* to regulate its transcription, which plays a role in the defense against WSSV infection (Li et al., 2015). In the present study, we found a (CT)*n* repeat in the 5′-UTR region of the *LvIRF* gene and designed primers to amplify the 5′-UTR including the (CT)*n* motif. PCR amplification of genomic DNA from 20 shrimp collected from diverse genealogies was performed and the amplified products had various lengths, ranging in size from 190 to 230 bp (**Figure 1A**). Sequencing results showed that the (CT)*n* microsatellite was polymorphic in this region, and could be further divided into a long (CT)*n* repeat and a short (CT)*n* repeat. Fourteen sequences, different in (CT)*n* motif number only, were chosen for further study. A pair wise alignment was then made using part of these sequences, such as (CT)_10+5_, (CT)_14+5_, and (CT)_18+7_ (**Figure 1B**).

### Estimating the Effect of (CT)*n* Motif Number Variation on *LvIRF* Expression

**Figure 2:**
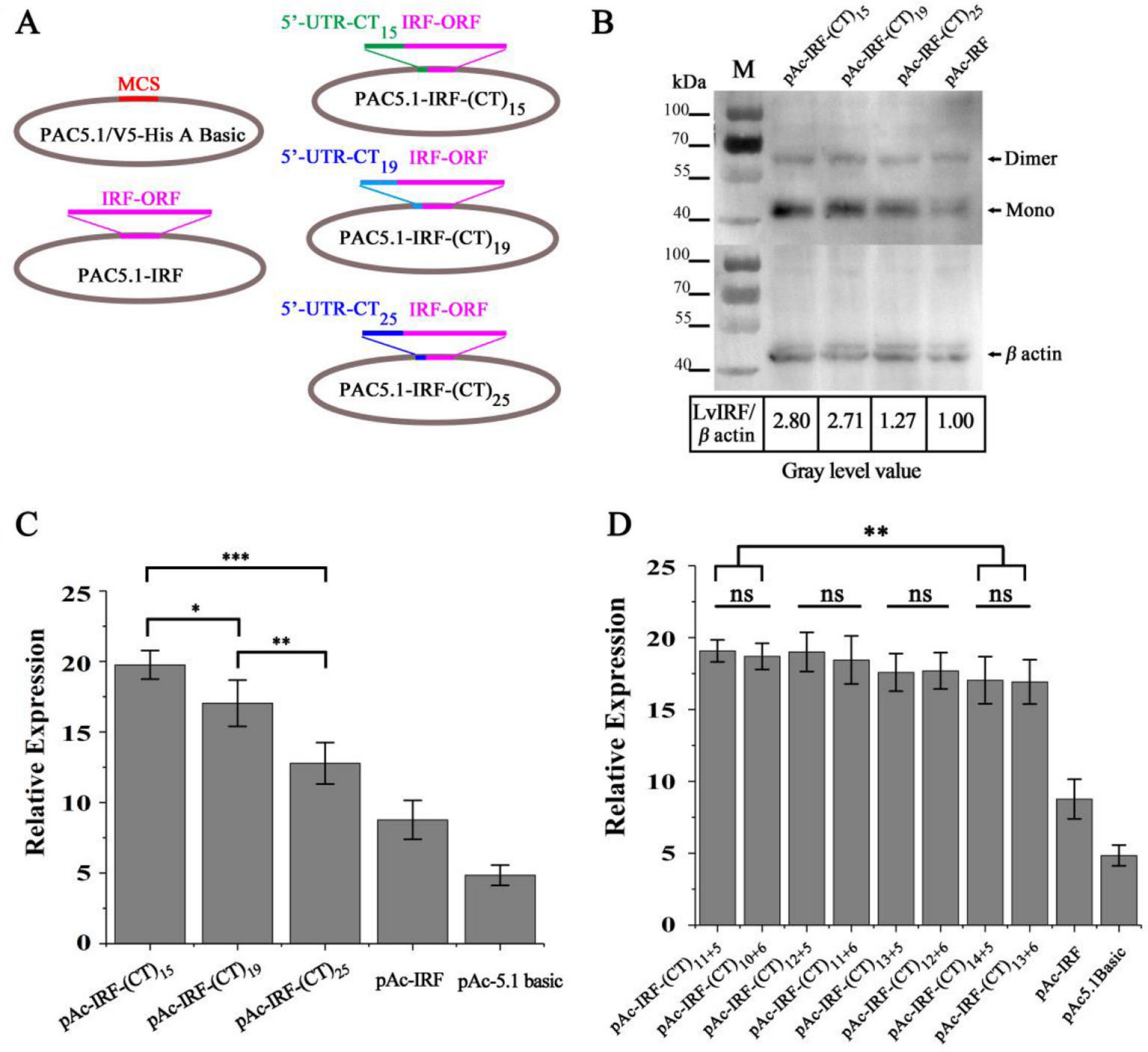
(CT)*n* repeats implicated in the expression of LvIRF. **(A)** Structures of 5′-UTRs harboring different (CT)*n* repeats and IRF coding sequence in pAc-5.1/His A vectors. **(B)** Western blots of LvIRF expression driven by different (CT)*n* repeat numbers. Gray level value ratios of LvIRF and *β*-actin in table, and the expression level from cells with pAc-IRF was set as the baseline (1.0). The ratio of LvIRF and *β*-actin with (CT)_15_ was twice as much as that with (CT)_25_. (C-D) Dual-luciferase reporter assays of LvIRF expression, **(C)** LvIRF expression with different total numbers of (CT)n repeats, **(D)** LvIRF expression with same total numbers of (CT)*n* repeats but different numbers of the two distinct (CT)*n* repeats. LvIRF expression with (CT)_15_ was 1.2-fold higher than that with pAc-IRF-(CT)_19_ (*p* < 0.05) and 1.5-fold higher than that with pAc-IRF-(CT)_25_ (*p* < 0.001). The pAc-IRF-(CT)_11+5_ and pAc-IRF-(CT)_10+6_ transfected cells showed no significant differences on the promoter activities of *LvVago4*, similar to the other three groups. *p < 0.05, **p < 0.01, ***p < 0.001, ns, not significant.

To investigate whether the variable numbers of (CT)*n* motif in the 5′-UTR of *LvIRF* are implicated with its expression, dual-luciferase reporter analysis in *Drosophila* S2 cells was carried out. The 5′-UTRs of the *LvIRF* gene harboring various (CT)*n* motifs were cloned into pAc-5.1-His/V5 A to generate pAc-IRF-(CT)*n*-V5 vectors for protein expression (**Figure 2A**). Three vectors, including 15, 19, and 25 (CT)*n* repeats, were constructed and transfected into S2 cells. The pAc-IRF-V5 and pAc-5.1-His/V5 A empty vector were also transfected into S2 cells as controls. To estimate the effect of (CT)*n* motif number variation on *LvIRF* expression, Western blotting was then performed. The results showed that the ratio of *LvIRF* and *β*-*actin* with (CT)_15_ was twice as large as that with (CT)_25_ in terms of gray-level values (**Figure 2B**). Previous reports have shown that the promoter of *LvVago4* contains a conserved IRF binding motif that was confirmed to be targeted by LvIRF (Li et al., 2015). To further confirm the above results (**Figure 2B**), dual luciferase reporter assays were performed using luciferase-expressing vectors containing the *LvVago4* promoter (**Figure 2C**). Maximal expression of *LvIRF* was observed in cells transfected with pAc-IRF-(CT)_15_, which was 1.5-fold higher than in cells with pAc-IRF-(CT)_25_ (*p* < 0.001), and 2.3-fold higher than in cells with pAc-IRF-V5 (*p* < 0.001). Considering that the (CT)*n* repeat is composed of two parts, we explored whether the different numbers of the two (CT)*n* repeats were implicated in *LvIRF* expression. We observed that the pAc-IRF-(CT)_11+5_ and pAc-IRF-(CT)_10+6_ showed no significant differences on the promoter activities of *LvVago4*. Similar results were observed in three other groups that contained a total of 17, 18, and 19 repeats, respectively (**Figure 2D**). Taken together, these results revealed that the expression of *LvIRF* was only affected by the total number of (CT)*n* copies.

### Specific (CT)*n* Microsatellite Genotypes Were Associated With WSSV-Resistance

**Figure 3:**
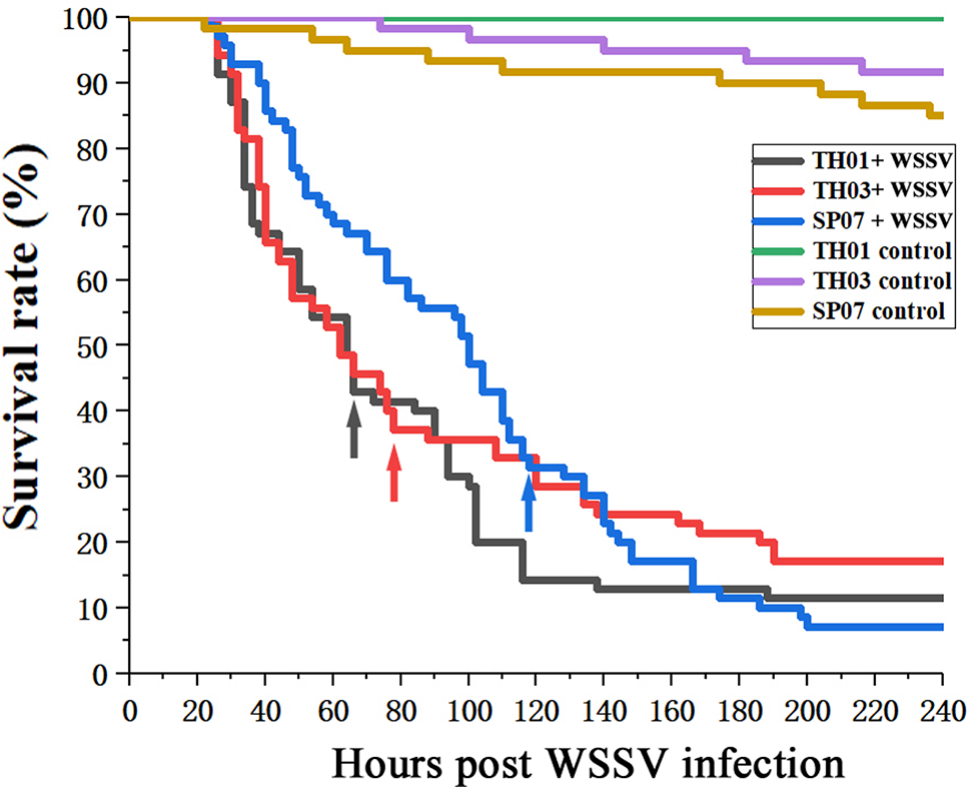
Survival rate curves for TH01, TH03 and SP07 shrimp. Three shrimp populations from different sources; TH01 from Vietnam, TH03 from China, and SP07 from Saipan. Arrows used to indicate the mortality peak times for the Sus. group (died before mortality peaks) and Res. group (died after mortality peaks).

**Figure 4:**
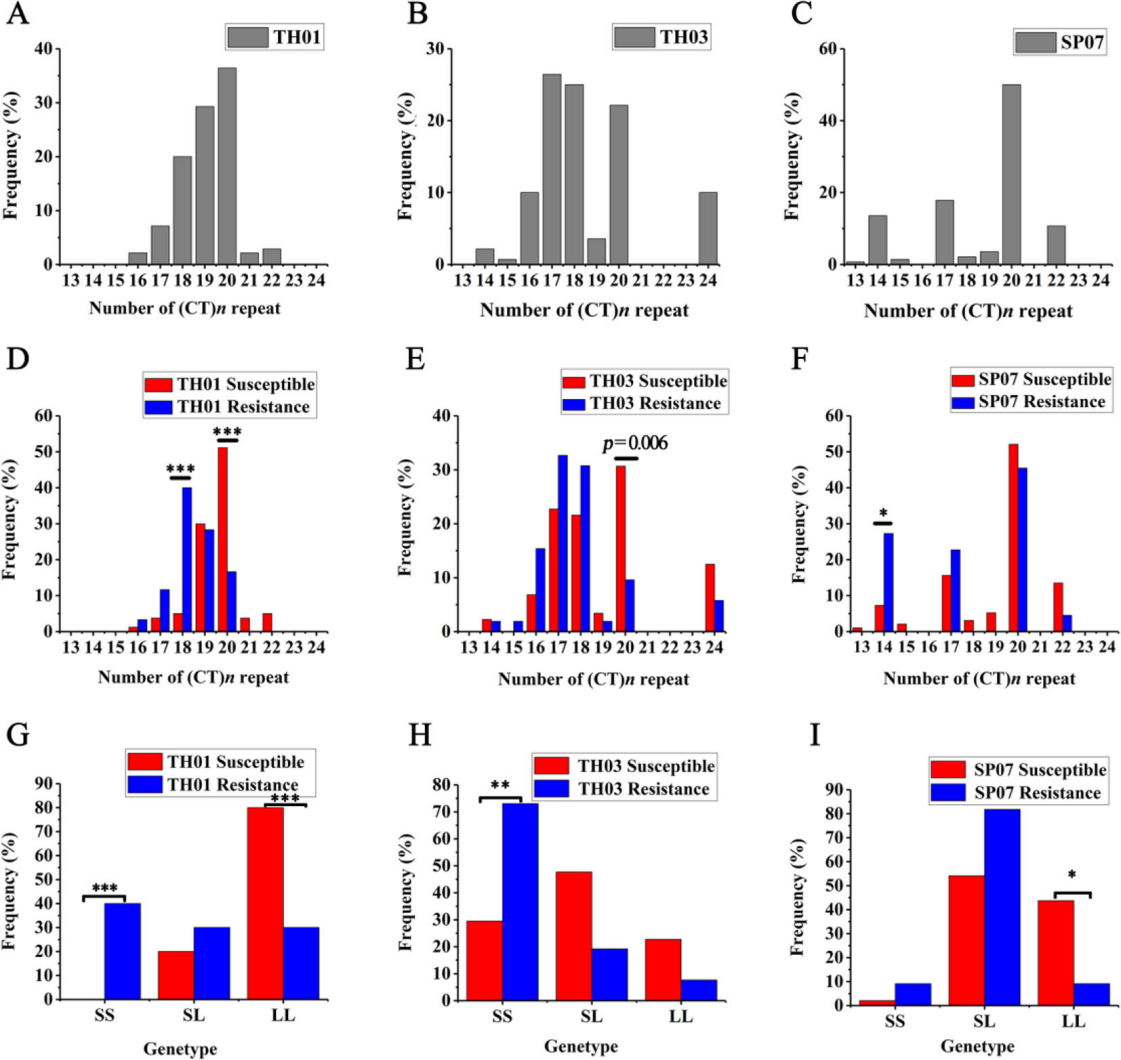
(CT)*n* microsatellite polymorphism allele distribution and genotype frequency in three different shrimp populations. **(A**–**C)** The allelic distribution in three population: TH01 **(A)**, TH03 **(B)** and SP07 **(C)**. **(D**–**F)** The allelic distribution in two groups of the three population (Fisher exact test: *p = 0.0042). **(G**–**I)** The genotype frequency in two groups of the three populations (Fisher exact test: *p = 0.0167). Associated with WSSV resistance, the TH01 and TH03 populations showed a significant difference in the S/S genotype, and the L/L genotype show a significant difference and was associated with WSSV susceptible in the SP07 population. *p < 0.05, **p < 0.01, ***p < 0.001.

According to the survival rate curve, we chose the mortality peaks (**Figure 3**, arrow) as the cut-off points for dividing each population of shrimp into two groups: WSSV-susceptible (Sus., died before mortality peaks) and WSSV-resistant (Res., died after mortality peaks) (**Table 2**). This phenotype was then genotyped in all three shrimp populations (**Figure 4**). The allelic distribution profile for each population was different. The number of (CT)*n* repeats in TH01 were skewed towards 18, 19, or 20 (**Figure 4A**), while TH03 was rich in 17, 18, or 20 (**Figure 4B**), and SP07 had an isolated peak at 20 (**Figure 4C**). The large difference in this allelic distribution indicated that the three populations used in this study had different genetic backgrounds, and showed a large degree of genetic stratification.

**Table 2:** Timing of mortality peaks and numbers of shrimp in each group.

Sample	Time/h	WSSV-Susceptible	WSSV-Resistant
TH01	66	40	30
TH03	78	44	26
SP07	118	48	22

Moreover, there was a significant difference when comparing the allelic distribution between Sus. group and Res. groups. In the TH01 population, the (CT)_18_ genotype was enriched in the Res. group associated with WSSV resistance, and showed a very significant difference with the Sus. Group (Fisher exact test: *p* < 0.00008, OR = 12.667, 95% CI = 4.09 - 39.225) (**Figure 4D**). On the contrary, the (CT)_20_ genotype was enriched in the Sus. Group (Fisher exact test: *p* = 0.00004, OR = 0.19, 95% CI = 0.085 - 0.427) (**Figure 4D**). In the SP07 population, the (CT)_14_ genotype was enriched in the Res. group (Fisher exact test: *p* = 0.0022, OR = 4.768, 95% CI = 1.726 - 13.168) (**Figure 4F**). In the TH03 population, the (CT)_20_ genotype was enriched in the Sus. Group (Fisher exact test: *p* = 0.0061, OR = 0.24, 95% CI = 0.086 - 0.671), although this difference was only nominally significant (**Figure 4E**).

Since the (CT)*n* repeat numbers ranged from 13 to 24 in the three populations, the repeat length cut-offs for allelic categorization were defined as ≤18 repeats for short (S) alleles and >18 repeats for long (L) alleles. Following allele classification, we could attribute to each individual one of three bi-allelic genotypes: S/S, S/L, or L/L. Genotype distribution frequencies were then determined and are shown for each population in **Figures 4G–I**. Associated with WSSV resistance, the TH01 population showed a very significant difference in the S/S genotype (Fisher exact test: *p* < 0.0003, OR = 28.053, 95% CI = 3.417 - 230.33) and the L/L genotype (Fisher exact test: *p* < 0.0003, OR = 0.107, 95% CI = 0.036 - 0.322). Similar to the TH01 population, the S/S genotype associated with WSSV resistance showed a significant difference in the TH03 population (Fisher exact test: *p* = 0.0005, OR = 6.476, 95% CI = 2.194 - 19.095), while the L/L genotype showed a significant difference in the SP07 population (Fisher exact test: *p* = 0.0054, OR = 0.129, 95% CI = 0.027 - 0.613).

Allelic distribution showed that S alleles tended to be enriched in Res. groups and L alleles tended to Sus. groups. We then compared the bi-allelic genotype distribution between the Sus. and Res. groups, and shrimp with the S/S genotype in the 5′-UTR of the *LvIRF* gene were resistant to WSSV, while those with L/L were susceptible to WSSV. The above results suggested that the shrimp with a short (CT)*n* repeat in the 5′-UTR of *LvIRF* were more resistant to WSSV than those with longer (CT)*n* repeats.

### Short CT Microsatellite Genotypes Improve the Resistance of Shrimp Populations to WSSV

**Figure 5:**
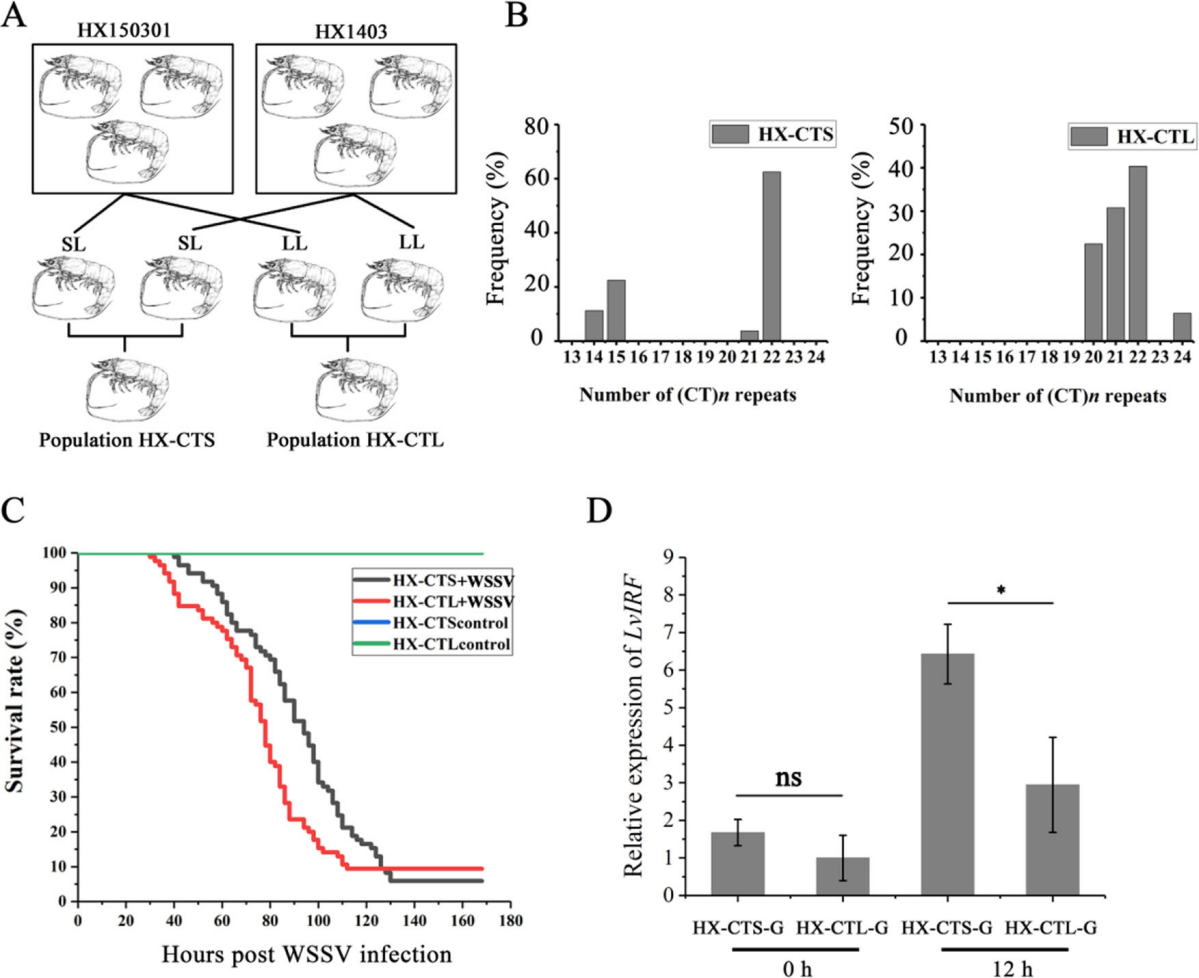
WSSV challenge in selective breeding offspring. **(A)** Schematic representation of the selective generations. **(B)** The allelic distribution in two populations, HX-CTS and HX-CTL. **(C)** The survival rate curves of HX-CTS and HX-CTL. **(D)** Quantitative RT-PCR was performed in triplicate for each sample using the EF-1α gene as an internal control by the Livak (2^−△△CT^) method. Expression levels in gill were used to determine the mean fold change (means ± SD, *n* = 5), and the expression level of the HX-CTL population at 0 h was used as a baseline (1.0). 12 h after infection with WSSV, LvIRF expression in HX-CTS was 2.1-fold higher than that of HX-CTL 12 h after infection with WSSV. *p < 0.05, ns, not significant.

Based on our breeding scheme illustrated in **Figure 5A**, we obtained two new shrimp populations. HX-CTS, containing short (CT)*n* repeats in the 5′-UTR of *LvIRF*, and HX-CTL, which only contained long (CT)*n* repeats. **Figure 5B** shows the allelic distribution in these two populations, which were as expected, and the HX-CTS population contained the (CT)_14_ and (CT)_15_ genotypes, while the HX-CTL population did not. The survival rate curve after WSSV infection is shown in **Figure 5C**. The survival rate curves of the two populations could be easily separated, as the HX-CTS mortality rate reached 50% 16 h later than the HX-CTL population. We also measured the *LvIRF* gene expression in gills by quantitative RT-PCR (**Figure 5D**). There was no difference at 0 h, but after 12 h of WSSV infection, the expression of *LvIRF* in the HX-CTS population was twice that of the HX-CTL population, which was a significant difference (*p* = 0.02). These results suggested that shrimp containing a smaller number of (CT)*n* repeats in the 5′-UTR of *LvIRF* were more resistant to WSSV infection, thereby improving their survival rate in this assay.

## Discussion

Our study examined the role of an SSR with a (CT)*n* motif in the 5′-UTR, as well as its repeat length variation, in the transcriptional regulation of the *IRF* gene of *L. vannamei* and the association of its genetic variations with WSSV resistance. Microsatellite repeats, especially (CT)*n* motifs, exist in abundance in the 5′-UTRs of several eukaryotic genes (Bhattacharyya et al., 2004; Mohammadparast et al., 2014; Zhao et al., 2014). The microsatellites are present in the vicinity of TSSs, indicating that they are likely involved in regulating target gene transcription (Castillo-Davis, 2005; Simpson and Ayyar, 2008). Our first goal was to investigate the highly polymorphic CT repeat located in the core promoter of the *LvIRF*-5’-UTR. Genotyping demonstrated a high heterogeneity of alleles, with lengths ranging from 13 to 24 repeats. In order to elucidate the role of (CT)*n* variation in the 5’-UTR of the *LvIRF* gene, we selected (CT)*n* variant alleles from different individuals and found that shrimp with smaller numbers of (CT)*n* repeats exhibited an enhanced tolerance to WSSV infection.

SSRs in the UTRs sequences or within promoter regions are thought to play a role in the regulation of gene expression. In *Catharanthus roseus*, *Tryptophan decarboxylase* (*Tdc*) gene expression is affected by the length of a (CT)*n* microsatellite in the 5’-UTR of this gene (Kumar and Bhatia, 2016). The chickpea *CaIMP* gene, with length variations of (CT)*n* repeat motifs present in the 5’-UTR, were differentially transcribed, and were shown to be associated with drought tolerance (Joshi-Saha and Reddy, 2015). Recently, a genome-wide survey of the contribution of microsatellites to gene expression in humans identified 2,060 significantly expressed microsatellites that were enriched in conserved regions, colocalize with regulatory elements, and may modulated certain histone modifications (Gymrek et al., 2016; Lieben, 2016). In our study, dual-luciferase reporter assays and Western blots clearly showed that the expression of the *LvIRF* gene was affected by the total of (CT)*n* repeat numbers. In particular, shorter repeats [(CT)_15_] showed higher expression of *LvIRF* than longer repeats [(CT)_25_].

IFNs are a group of secreted cytokines with activities that inhibit viral replication, and are able to regulate the functions of several types of immune cells. In mammals, type I and III IFNs exhibit significant antiviral activities, and are considered to be central to antiviral innate immunity (Levy et al., 2011). The IRF family is a group of transcription factors that plays critical roles in the activation of IFNs (Ikushima et al., 2013). *LvIRF* is an IRF in crustaceans, with functions similar to mammalian IRFs. *LvIRF* mediates the IRF-Vago-JAK/STAT pathway in shrimp, and has been shown to inhibit viral (WSSV) replication, analogous to the IRF-IFN-JAK/STAT pathway in vertebrates (Li et al., 2015). We used shrimp populations with allelic distribution differences to analyze the relationship between *LvIRF* genotype and WSSV resistance. Our results demonstrated that the *LvIRF* (CT)*n* repeat number was associated with WSSV resistance in shrimp. Smaller numbers of repeats showed significant resistance to WSSV, perhaps due to the higher *LvIRF* expression. We selected three populations of shrimp with different genetic backgrounds and allelic distributions of *LvIRF* to analyze the association between (CT)*n* repeats and WSSV resistance, and obtained similar results. This indicated that the regulation of *LvIRF* expression by an SSR with a (CT)*n* motif in the 5′-UTR was widespread in *L. vannamei*. Studies have shown that the IRF-IFN-JAK/STAT pathway is a broad-spectrum antiviral pathway in vertebrates (Thompson et al., 2014; Qin et al., 2016). *LvIRF* had similar functions to mammalian IRF, indicating that the IRF-Vago-JAK/STAT pathway may induce immune responses to a wide range of viruses, including WSSV. Microsatellite polymorphisms associated with disease and biological traits have also been reported. For example, more than 25 inherited human disorders have been shown to be caused by STRs (Sun et al., 2018), including FXS (Colak et al., 2014), Huntington’s disease (Wheeler et al., 2007), Friedreich’s ataxia (Shishkin et al., 2009), and idiopathic short stature (Dias et al., 2017).

We also used the microsatellite of *LvIRF* as a molecular marker to breed two populations of offspring, which contained different SSR genotypes and exhibited distinct tolerances to viral infection. The shrimp with small numbers of (CT)*n* repeats had a stronger antiviral immune response, as manifested by observation of an increased *LvIRF* gene expression and the prolonged survival time after WSSV infection. Thus, the identified SSR could be candidate marker for shrimp breeding for WSSV resistance. In fact, molecular markers have been widely reported in the breeding of several cash crops, including rice and wheat (Xue et al., 2018). For example, by analyzing the agronomic traits of rice varieties throughout the world, a number of molecular markers have been obtained for marketable traits, such as grain size: *GLW7/OsSPL13* (Si et al., 2016), chilling tolerance: *COLD1* (Ma et al., 2015), disease resistance: *Pigm* (Deng et al., 2017), and nitrate-use efficiency: *NITR1.1* (Hu et al., 2015). Some successful cases of molecular marker assisted breeding have also been reported in aquatic animals. For instance, using transcriptome sequencing analysis, eight IFN system genes were identified as anti-disease molecular markers for resistance breeding of gibel carp (Mou et al., 2018). The use of molecular markers has made shrimp designer breeding possible in the future.

In summary, we identified an SSR of a (CT)*n* repeat located in the 5′-UTR of *LvIRF* and demonstrated that the total length of these (CT)*n* -SSRs could influence the levels of gene expression. In addition, we also demonstrated that this SSR was associated with WSSV resistance. Our results provide some insights into how this SSR could be used as a molecular marker in breeding of WSSV-resistant shrimp. Further work needs to be done to investigate the feasibility of other molecular markers in shrimp breeding.

## Data Availability Statement

All reagents and experimental data are available within the article or supplementary information or from corresponding author upon reasonable request.

## Author Contributions

CL and JH conceived and designed the experiments. BY, HW, PZ, and SW performed the experiments and analyzed data. CL and BY wrote the draft manuscript. CL was responsible for forming the hypothesis, project development, data coordination, and writing, finalizing, and submitting the manuscript. All authors discussed the results and approved the final version.

## Funding

This research was supported by National Key Research and Development Program of China (2018YFD0900502/5), the Science and Technology Planning Project of Guangdong Province (2018B020204001); National Natural Science Foundation of China (31772883); Guangdong Natural Science Funds for Distinguished Young Scholars (2016A030306041); Tip-Top Scientific and Technical Innovative Youth Talents of Guangdong Special Support Program (No. 2016TQ03N504), and Fundamental Research Funds for the Central Universities (17lgpy62). The funders had no role in study design, data collection and analysis, decision to publish, or preparation of the manuscript.

## Conflict of Interest

The authors declare that the research was conducted in the absence of any commercial or financial relationships that could be construed as a potential conflict of interest.

The reviewer JX declared a shared affiliation, with no collaboration, with the authors to the handling editor at time of review.
